# Impact of Interactions Between Zn(II) and Selenites in an Aquatic Environment on the Accumulation of Se and Zn in a Fungal Cell

**DOI:** 10.3390/molecules30143015

**Published:** 2025-07-18

**Authors:** Małgorzata Kałucka, Piotr Podsadni, Agnieszka Szczepańska, Eliza Malinowska, Anna Błażewicz, Jadwiga Turło

**Affiliations:** 1 Department of Drug Technology and Pharmaceutical Biotechnology, Medical University of Warsaw, 61 Żwirki i Wigury Street, 02-091 Warsaw, Poland; malgorzata.kalucka@wum.edu.pl (M.K.); piotr.podsadni@wum.edu.pl (P.P.); agnieszka.szczepanska@wum.edu.pl (A.S.); eliza.malinowska@wum.edu.pl (E.M.); 2 Department of Pathobiochemistry and Interdisciplinary Applications of Ion Chromatography, Medical University of Lublin, 19 Chodźki Street, 20-093 Lublin, Poland; anna.blazewicz@umlub.pl

**Keywords:** zinc, selenite, *Lentinula edodes*, biosorption, zinc–selenite interactions

## Abstract

Our attempts to obtain a new mushroom-derived immunostimulatory preparation containing organically bound selenium and zinc have focused on the interactions between selenites and zinc(II) in liquid culture media and their effects on transport into the mushroom cell. Previously, we found that, even if Zn^2+^ and SeO_3_^2−^ concentrations in the liquid medium are not high enough to precipitate ZnSeO_3_, the accumulation of selenium in the presence of zinc, and zinc in the presence of selenites, significantly dropped. This effect was more dependent on the molar ratio of ions in the medium than on the concentration values. We hypothesized that the formation of zinc–selenite soluble complexes with charges depending on the ion concentration ratio in the aquatic environment affects the first stage of ion transport into the fungal cell—biosorption. To verify this, we found the zinc–selenite molar ratio at which the complexes of the highest stability are formed, examined the influence of the molar ratio of ions in the medium on the concentration of Zn and Se in the mushroom cell wall, and investigated the correlation between the concentration of selenites not bound in complex compounds and the Se concentration in the cell wall. The results indicate that the molar fraction of Zn(II) in a liquid medium in the range of 0.5–0.6 promotes the formation of the most stable complexes. At the same time, it significantly reduces the percentage of free selenites in the medium and most strongly inhibits the biosorption process of both zinc and selenium.

## 1. Introduction

Fungi have a unique ability to accumulate trace elements. This ability can be used in the development of various technologies, e.g., in the bioremediation of soil and water contaminated with heavy metals or, conversely, the enrichment of mushroom-derived functional foods with bioelements [1,2,3,4]. Submerged cultures of *Lentinula edodes*, an edible and medicinal mushroom, have been used in our previous research both to examine the influence of environmental conditions on the absorption of microelements in fungi [5] and to obtain new immunomodulatory preparations rich in selenium and zinc [6,7,8].

*Lentinula edodes (Berk.) Pegl*., known as Shiitake, is one of the most valuable medicinal mushrooms, renowned for thousands of years both as a food and medicine. This mushroom contains all of the essential amino acids in its proteins and significant quantities of vitamins D, C, B_1_, B_2_, PP, and B_12_ [9,10,11,12]. A highly purified polysaccharide fraction extracted from *L. edodes* fruitbodies, or mycelium–lentinan, is an approved drug used in cancer treatment in several countries, as well as in AIDS research. It may be used as a non-invasive form of cancer treatment due to its ability to induce a non-specific immune system response against cancer cells. This capacity is also evident against viral and bacterial infections and inflammation. Various substances with immunopotentiating, antiatherosclerotic, antiviral, hepatoprotective, and hypercholesterolemic effects have also been isolated from *L. edodes* [13,14,15,16,17]. The strong immunomodulatory properties of the polysaccharides contained in the cell wall of L. edodes (lentinan and others) allowed us to hypothesize about possible synergy with the similar activity of selenium and zinc compounds.

Recent studies by independent research groups have increasingly focused on the role of fungi in the bioaccumulation of trace elements such as selenium and zinc from environmental and nutritional perspectives. For example, in *Pleurotus eryngii*, co-supplementation with zinc and selenium was found to significantly increase the accumulation levels of both elements while also enhancing antioxidant activity and relevant biochemical markers in the biomass [18]. Similarly, *Flammulina velutipes* exhibited dramatic synergistic effects when cultured with both elements, showing an 85- to 88-fold increase in selenium accumulation relative to single-element supplementation [19]. These findings highlight the importance of not only the presence of Zn(II) and Se(IV) but also their molar ratios and chemical forms, which appear to govern uptake efficiency. This concept is further supported by microbial systems such as *Pseudomonas putida*, where the speciation of selenium strongly influences its transformation into nano-Se particles, illustrating the broader relevance of physicochemical controls in bioaccumulation processes [20].

Beyond fungal models, the synergistic effects of selenium and zinc co-supplementation have also been described in plant and animal studies. In wheat (*Triticum aestivum*), foliar co-application using Zn and Se increases the concentrations of both elements in grains, though sometimes at the cost of reduced Se uptake compared with selenium-alone treatments, underscoring the importance of precise ratio control [21]. In animal models of metabolic disorders, combined supplementation improved antioxidant enzyme activity and lipid metabolism [22,23], while preliminary clinical trials suggest functional benefits in humans, though the evidence remains inconsistent [24].

Together, these data reinforce the rationale for studying Zn–Se interactions at the molecular and physicochemical levels, especially in fungal systems where accumulation pathways are particularly complex.

In our previous studies, we showed that the mycelium of *L. edodes* cultivated in a liquid medium accumulated selenium very effectively when this element was introduced to the culture medium in the form of sodium selenite [6]. Most of this element was transformed by the fungus into organic compounds, i.e., selenomethionine, selenocysteine, methylselenocysteine, and Se-containing polysaccharides [6,7,8,25,26,27]. Simultaneously, in the same mushroom species, we examined the accumulation of zinc introduced to the liquid medium in the form of zinc bromide [28]. This process was also very effective: the total concentration of zinc in the mycelial dry weight was high, with about 86% of zinc accumulated intracellularly [28].

Our attempts to obtain a new type of dietary supplement containing both selenium and zinc, two micronutrients necessary for the functioning of the immune system, extended our interest in the simultaneous accumulation of these elements through mycelia growth in liquid media rich in selenite and zinc(II) ions [29]. In a series of experiments, we found that the ratio of molar Zn(II) and SeO_3_^2−^ ion concentrations in the medium was variable, but the concentrations were not high enough to precipitate ZnSeO_3_. The accumulation of selenium in the presence of zinc, and zinc in the presence of selenium, strongly decreased in comparison with the accumulation of that ion alone [29]. The most interesting finding was that this effect depended more on the molar ratio of ions in the culture medium than on the ion concentrations. The strongest indication came from the results obtained at a Zn^2+^-to-SeO_3_^2−^ concentration ratio of 1:1 (0.2 mM:0.2 mM) [29]. To explain this phenomenon, we hypothesized that interactions between ions in a liquid medium may be responsible for this. Since the precipitation of ZnSeO_3_ was not possible at such low ion concentrations in the solution, we assumed that the mutual inhibition of zinc and selenium accumulation resulted from the formation of soluble Zn(II)–selenite complexes in the culture medium. The formation of Zn(II)–selenite complex compounds with solubility, strength, and charge values depending on the molar ratio of ions—in some cases organically templated—has been described by numerous researchers [30,31,32,33,34,35]. We assumed that ion complexation in the water medium would impair interactions with the fungal cell wall, especially when the stability of the formed complex compound is high. The accumulation of ions from the substrate in fungi comprises three phases: biosorption on the surface of the fungal cell wall, intracellular uptake, and chemical transformation [36,37]. We assumed that the formation of zinc–selenite complexes of different charges would inhibit the first stage of the accumulation process, i.e., biosorption, which involves binding ions to the cell wall. To verify our hypothesis, we decided to check whether zinc–selenite complexes are formed in the culture medium and how their stability depends on the molar ratio of ions in the medium. In the next step, we wanted to check which of the stages of zinc and selenium accumulation (biosorption or intracellular absorption) depended on the interaction between Zn(II) and selenites. We investigated the latter by checking whether the concentration of selenium and zinc in the isolated cell walls depends on the formation of zinc–selenite complexes and the concentration of uncomplexed ions in the culture medium.

## 2. Results

### 2.1. Impact of SeO_3_^2−^/Zn^2+^ Molar Ratio in Culture Medium on the Concentration of Se and Zn in the Mushroom Cell Wall

Since the first stage of microelement transport into the fungal cell involves binding to the cell wall (biosorption), determining the concentration in the isolated cell wall can be used to assess the ion biosorption process. The results of our previous studies show that the molar ratio of the selenites and zinc(II) in the culture medium is an important factor in selenium and zinc absorption in fungal cells [29]. In other studies, a similar relationship was found for the isolated fungal cell walls (Table 1).

As described in the Section 5, the mycelial cultures of the fungus *Lentinula edodes* were grown in liquid media supplemented with SeO_3_^2−^ and Zn^2+^ in the following concentration ratios (mM): 0:0, 0.2:0, 0.2:0.2, 0.2:0.4, and 0.2:0.8 and 0:0.2, 0.2:0.2, 0.4:0.2, and 0.8:0.2. Thus, the concentration ratios of Se and Zn ions in the solution were 1:1, 1:2, 1:4, 2:1, and 4:1. The ion concentrations were so low that ZnSeO_3_ precipitation was impossible (the solubility product was not exceeded). The unenriched medium (0:0) and media enriched with only one of the ions (0.2:0 and 0:0.2 mM) were the controls.

**Figure 1:**
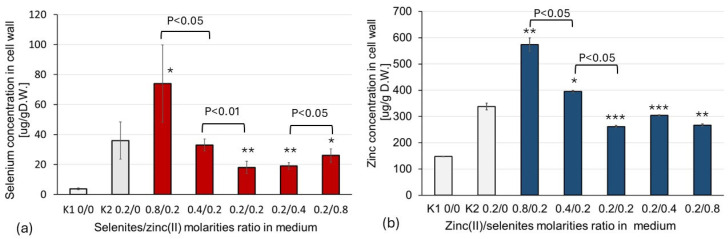
The effect of the Zn(II)/selenite molar ratio in the medium on the biosorption of Se and Zn: (**a**) selenium concentration in the cell wall as a function of the selenite and zinc(II) molar concentration ratio in the cultivation medium; (**b**) zinc concentration in the cell wall as a function of zinc(II) and selenite molar ratio in the cultivation medium. The data represent the mean ± S.D. of five observations. The following levels of significance versus control (K_1_) were defined: *p* < 0.05 *; *p* < 0.01 **; and versus control (K_2_), *p* < 0.05 ***.

The selenium content in the cell wall samples decreased significantly, by fourfold (*p* < 0.05), when the selenite–zinc(II) molar ratio in the medium decreased from 4:1 to 1:1 (Figure 1a). When the molar ratio of selenite–zinc(II) in the culture medium rose from 1:1 to 1:4, the Se content in the mycelial cell walls slightly increased (*p* < 0.05) (Figure 1a). A similar effect was observed for the zinc content in the fungal cell walls when the zinc(II)—selenite molar ratio in the medium decreased from 4:1 to 1:1; the amount of absorbed zinc decreased by ~50% (*p* < 0.05) (Figure 1b). However, in the case of zinc, the excess of selenites (molar ratio; Zn(II): SeO_3_^2−^, 1:2 and 1:4) had almost the same effect on zinc biosorption as a molar ratio of 1:1 (Figure 1b). Thus, the decrease in the biosorption of both selenites and Zn(II) was most evident when the molar ratio of zinc ions and selenites was close to 1:1.

**Figure 2:**
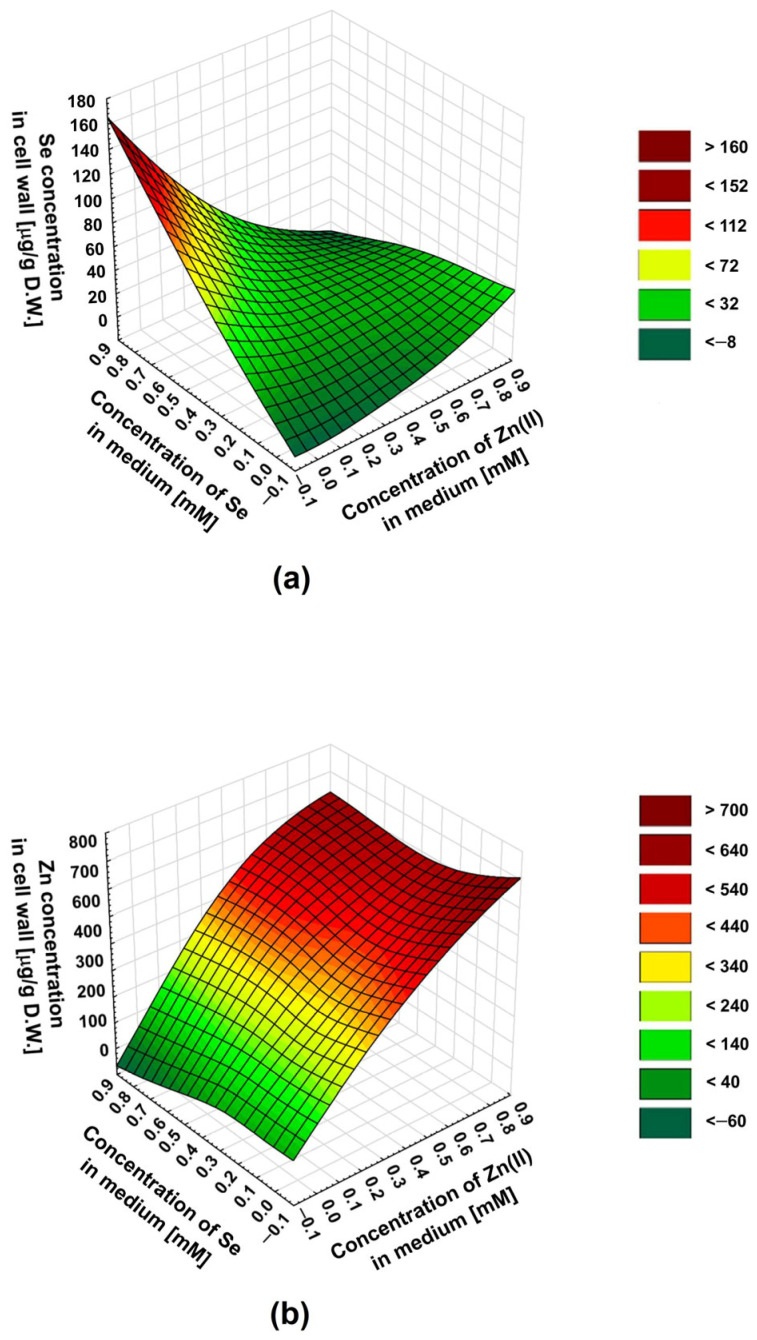
A 3D surface plot illustrating the relationship between (**a**) Se concentration in the cell wall, (**b**) Zn concentration in the cell wall, and two variables: Zn(II) and selenite concentrations in the culture medium.

To predict the influence of the selenite–zinc(II) concentration ratios on the culture medium—other than those we tested on the biosorption of these ions—we used the statistical method: the response surface methodology (RSM). Regression analysis uses a sequence of designed experiments to obtain an optimal response. In our experiments, we wanted to predict the optimal combination of zinc(II) and selenite concentrations in the medium for the most effective simultaneous biosorption. Thus, we decided to use the RSM to explore the relationship between two variables (concentrations of Zn(II) and selenites) and one response variable (zinc or selenium concentration in the cell wall). The differences in the inhibition of Zn(II) and selenite binding to the cell wall in the presence of an excess of the latter are well illustrated in three-dimensional surface plots (Figure 2a,b).

For the selenites, the maximum inhibition of the biosorption was at a selenite–zinc molar ratio in the medium of about 1:1. An increasing excess of zinc(II) in the medium does not decrease selenite biosorption—a slight increase in selenium concentration in the cell wall was observed. This is indicated by the selenite–zinc concentration ratio resulting in the lowest selenium content in the cell wall, for a given selenite concentration in the medium (a characteristic “valley” on the surface plot—Figure 2a). In the case of zinc, the maximum inhibition of biosorption also occurs at a selenite–zinc molar ratio in the medium of about 1:1; however, this effect was observed only for low zinc(II) concentrations in the medium (0.2–0.4 mM). For higher concentrations of zinc(II) (0.5–0.8 mM), the maximum inhibition of biosorption was observed for a 2-fold excess of these ions in relation to selenites. This was indicated by the zinc–selenite concentration ratio in the medium, resulting in the lowest zinc concentration in the cell wall for a given zinc concentration in the medium (a characteristic “valley” on the surface plot, less visible in this case—Figure 2b). The differences in the effect of the concentration ratio of selenite and zinc(II) in the medium on the biosorption of these two ions are an interesting phenomenon and probably result from different types of cation and anion interactions with the cell wall.

### 2.2. Determination of the Composition and Stability of Zn(II)-Selenite Complexes in the Liquid Culture Medium Using the Method of Continuous Variations (Job’s Method)

**Figure 3:**
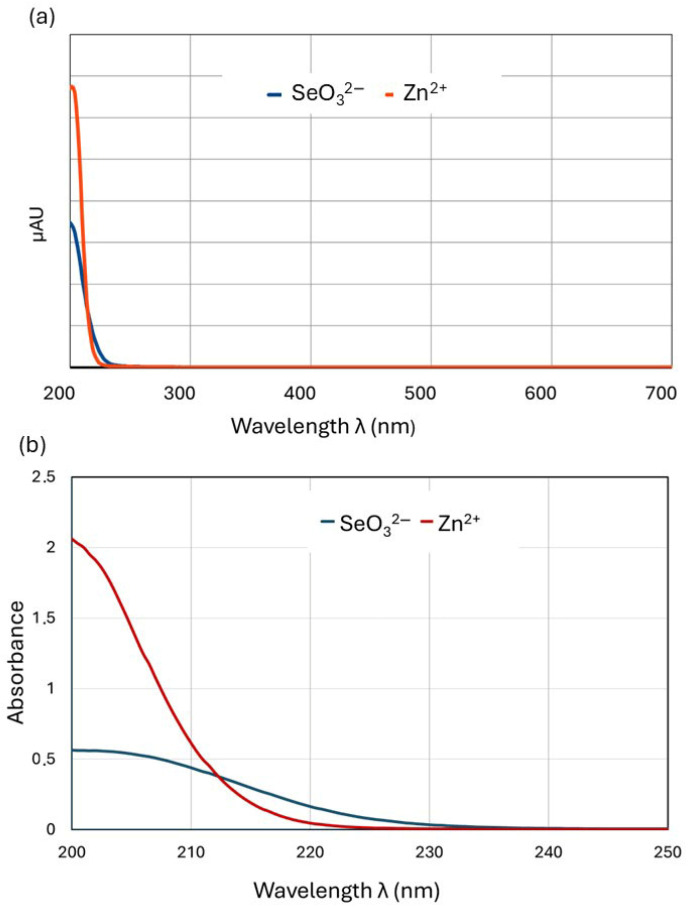
Absorbance versus the wavelength graph for 0.4 mM solutions of SeO_3_^2−^ and Zn^2+^ recorded using (**a**) a Shimadzu UHPLC liquid chromatograph with diode-array detector (λ range: 200–700 nm) and (**b**) a UV–Vis Mini 1240 Shimadzu spectrometer (λ range 200–250 nm).

The results described above indicate that the molar ratio of zinc(II) and selenites in the culture medium clearly affects the binding of these ions to the fungal cell wall (biosorption). We assume that the phenomenon that inhibits the biosorption process is the complexation of zinc with selenites, which depends on the concentration ratio. To verify this hypothesis, we decided to detect the existence, strength, and stoichiometry of such associations using the method of continuous variations (MCV or Job’s method). Job’s method relies on mixing reactant (central ion, M, and ligand, L) stock solutions of equal molar concentrations in different volume ratios while keeping the total volume constant. Then, the absorbance (A) of the solution containing the central ion and the ligand in different mutual ratios is measured. The peak of absorbance versus the mole fraction (χ) of one of the components (Job plot) identifies the ratio of the ligand (L) to the metal (M) at stoichiometric proportions and thus determines the formula of the coordination complex (M_n_L_m_) [38]. Instead of the more commonly used UV–Vis absorbance [39], any property that correlates linearly with the M_n_L_m_ concentration suffices, including conductivity [40], permittivity [41], relaxivity [42], NMR spectroscopy [43,44], calorimetry [45], circular dichroism [46,47,48], circular polarized luminescence [49,50], gravimetric titration [51], melting point depression [52], and others. For solutions with both selenite and Zn(II) ions, we found a linear absorbance dependence measured at 215 nm in the solution concentration, so it was possible to plot Job’s diagrams based on absorbance measurements. To conduct the experiments under conditions corresponding to the processes taking place in the *L. edodes* liquid medium, we selected a stock solution concentration (0.4 mmol/L) within the range of concentrations used to supplement culture media (0.2–0.8 mmol/L). The experiments on the formation of zinc–selenite complexes were initially conducted in pure aqueous solutions but were then repeated in the liquid culture medium. The zinc–selenite complexes described in several studies have diverse structures, and the wavelength at which they exhibit maximum absorbance is impossible to predict; thus, we had to find the optimum wavelength experimentally. We scanned the 200–700 nm wavelength range using two methods involving a UV–Vis spectrophotometer (UV Mini-1240, Shimadzu, Kyoto, Japan) and a Shimadzu UHPLC chromatograph with a diode-array (DAD) detector. Using a quartz cuvette with a UV–Vis spectrophotometer allowed us to also scan the short-wave range. To confirm the results, the 200–700 nm range was scanned again using a diode array HPLC detector. The optimum absorbance was observed in a range of 200–220 nm (Figure 3).

**Figure 4:**
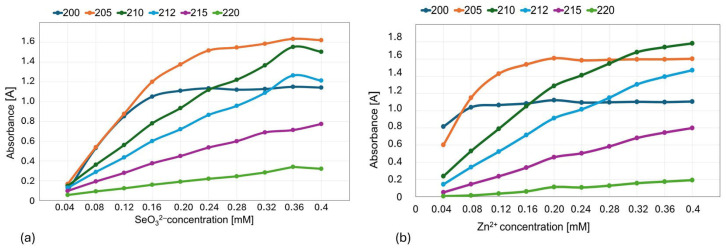
Absorbance versus concentration graph for (**a**) SeO_3_^2−^ and (**b**) Zn^2+^ ions at the tested wavelengths (200, 210, 212, 215, and 220 nm).

**Figure 5:**
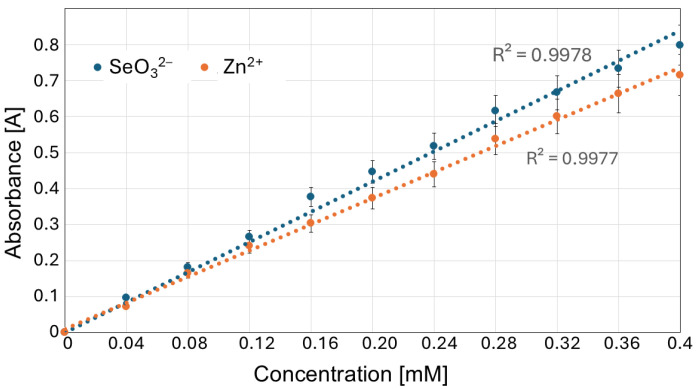
Absorbance versus concentration graph for SeO_3_^2−^ and Zn^2+^ at the 215 nm wavelength.

Out of the subsequently tested wavelengths (200, 210, 212, 215, 220; Figure 4), we selected 215 nm for further experiments, in which both Na_2_SeO_3_ and ZnBr solutions showed a linear absorbance–concentration relationship (Figure 5).

**Figure 6:**
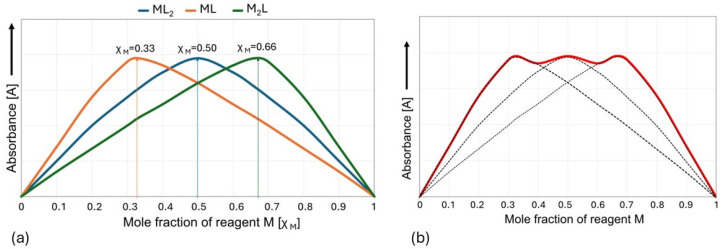
Job plot showing (**a**) 1:2, 1:1, and 2:1 binding stoichiometries for an M_n_L_m_ complex and (**b**) an example of overlapping peaks.

In practice, it is easiest to use the MCV for systems in which there is only one complex in the solution that predominates over all others under experimental conditions. For a hypothetical complexation reaction, nM + mL ⇋ MnLm, the maximum position of the Job plot at χ_M_ = 0.5 provides a 1:1 stoichiometry (m = n) ratio, a maximum at χ_M_ = 0.33 provides a 1:2 stoichiometry (m = 2n) ratio, a maximum at χ_M_ = 0.66 provides a 2:1 stoichiometry (n = 2m) ratio, etc. (Figure 6a). However, when more than one stable complex is formed, the graph may result from the superposition of two or more peaks and be more difficult to interpret [38,53,54,55,56,57] (Figure 6b).

**Figure 7:**
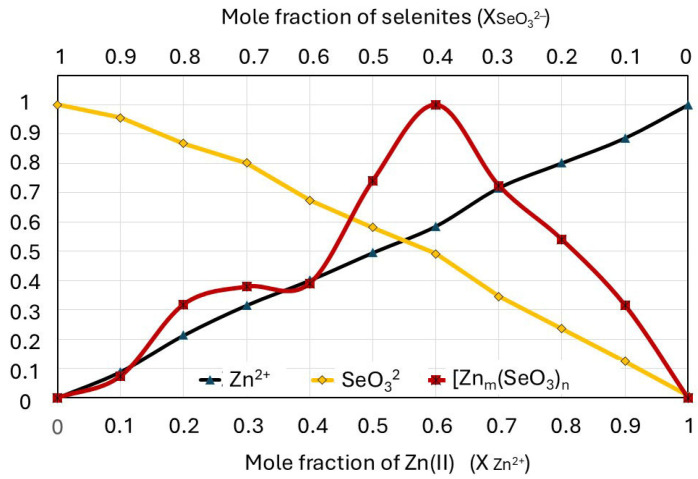
Job plot for complexation of zinc with selenites compared with curves for Zn^2+^ and SeO_3_^2−^ solutions of the same concentrations, as in the mixture.

A normalized Job plot for the selenite–zinc mixtures prepared according to the MCV is shown in Figure 7. As a reference, an absorbance–concentration relationship is noted for each component of the mixture (solutions of Na_2_SeO_3_ and ZnBr).

The concentration of each ion is in a range of 0–0.4 mmol/L, which corresponds to their mole fraction in the mixture (0–1). The Job plot indicates that only one complex predominates in the solution over all others (Figure 6a); furthermore, the graph shows a second smaller peak overlapping with the larger one (Figure 6b). The position of the maximum Job plot at χ_Zn(II)_ = 0.6 provides the 6:4 (3:2) stoichiometry, which corresponds to the hypothetical formula of the complex ion [Zn_3_(SeO_3_)_2_]^2+^. The maximum position of the smaller peak at χ_Zn(II)_ = 0.25 provides the 1:3 stoichiometry (0.25:0.75), which corresponds to the hypothetical formula of the complex ion [Zn(SeO_3_)_3_]^4−^. The molar ratios of zinc(II) and selenite ions in the complexes determined using Job’s diagram do not have to be their full formulas. Zinc(II) ions also form coordination bonds with water molecules in an aqueous environment, so their additional presence is highly probable.

According to the literature, zinc(II) may have a coordination number of four, five, or six, though four-coordinate complexes are most common [58]. To verify the unexpected course of the Job plot, the experiments were repeated at other wavelengths (220 and 210 nm) and using a high-performance liquid chromatograph with a diode-array detector instead of a UV–Vis spectrophotometer. For all repetitions and determination methods, the Job plot with two visible peaks remained unchanged.

**Figure 8:**
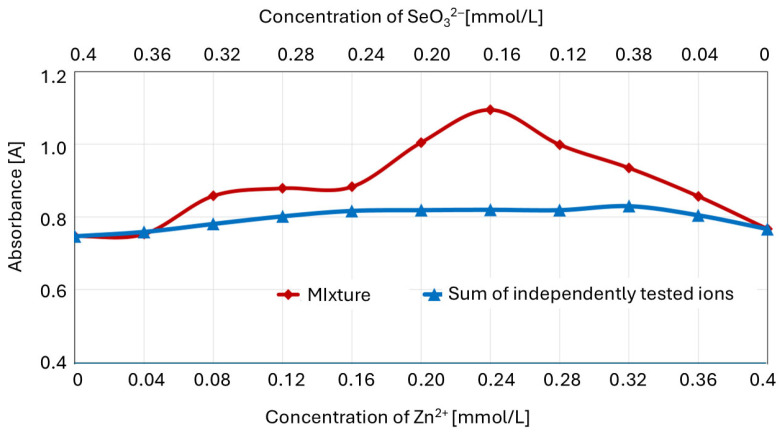
Absorbance of the Zn^2+^ and SeO_3_^2−^ mixture (Job plot) versus the sum of the separately determined ion absorbances.

To confirm the formation of the zinc–selenite complexes for certain ion concentration ratios, the Job plot was compared with the sum of the absorbances of separately determined ions (Figure 8).

For the zinc(II)–selenite concentration ratios in the mixture (expressed in millimoles per liter) of 0.08:0.32 (1:3) to 0.36:0.04 (9:1), there was a significant (*p* ≤ 0.05) difference between the absorbance of the ion mixture and the sum of the absorbances of the separately determined solutions of component ions.

**Figure 9:**
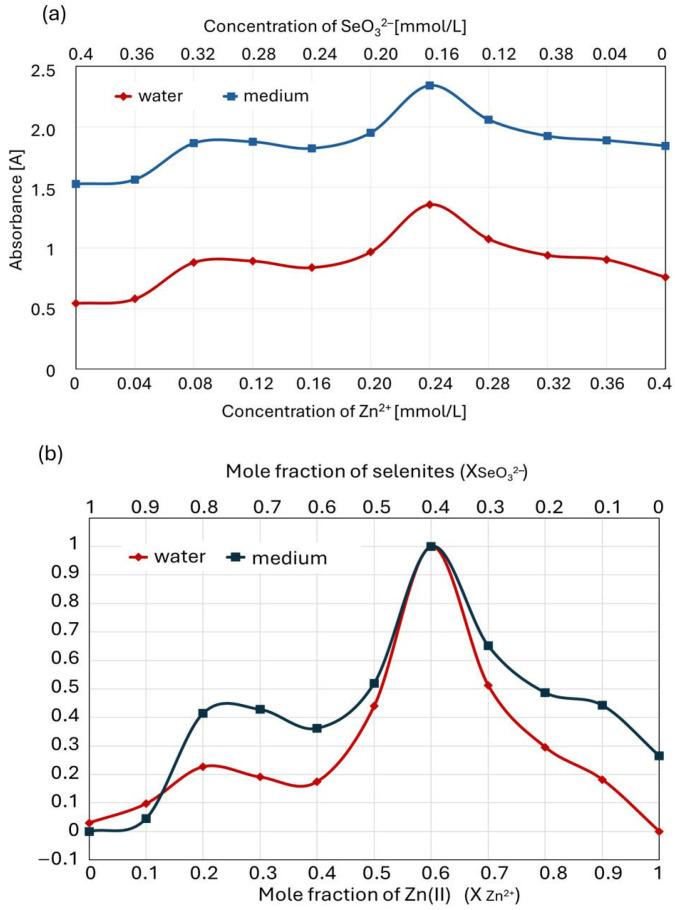
Comparison of Job plot for zinc complexation with selenite in pure aqueous solution and in the culture medium: (**a**) actual absorbance value; (**b**) normalized plots.

Since the liquid culture medium in which the complexation of zinc(II) with selenite ions takes place contains numerous components that may interact with these ions (e.g., amino acids), we repeated experiments analogous to those described above for the solutions comprising Zn(II) and selenites dissolved in a culture medium (instead of in pure water). Interestingly, the Job plot for zinc complexation with selenites in the liquid culture medium was consistent with the plot recorded for ion interactions in pure water. The total absorbance values of the solutions were slightly higher in this case due to the complex composition of the medium (in both cases, the measurements were performed with the same blank, water, while additional compounds absorbing at a wavelength of 215 nm were present) (Figure 9a). However, the course of the normalized Job plot was similar (Figure 9b). As in pure water solutions, the Job plot showed two maxima, with the higher peak at χ_Zn(II)_ = 0.6, providing a zinc–selenite concentration ratio of 6:4 (3:2); conversely, the maximum of the smaller peak at χ_Zn(II)_ = 0.25 provides a 1:3 stoichiometry ratio (0.25:0.75).

The Job plot of the culture medium indicates higher stability for the complexes formed at a zinc(II) mole fraction of 0.2–0.3 than in pure water. Furthermore, higher stability can be observed for the complexes in the medium at a zinc(II) mole fraction of 0.8–0.9. Since selenite–zinc complexes can also interact with other molecules present in the culture medium, e.g., amino acids and proteins containing amino groups, we put forward a hypothesis about the higher stability of such compounds. However, its verification requires separate studies.

In summary, the results obtained using Job’s method confirm our assumption that, depending on the molar ratio of zinc(II) and selenites in the culture medium, complex compounds with different charges and stability are formed, affecting the biosorption process of these ions.

### 2.3. The Effect of the Zn^2+^/SeO_3_^2−^ Molar Ratio in the Cultivation Medium on the Concentration of Non-Complexed SeO_3_^2−^ Ions

Since our assumption was that the formation of complex compounds by selenites and zinc(II) in the culture medium hampered the biosorption process, we decided to determine the concentrations of unbound (“free”) SeO_3_^2−^ ions in culture media and to examine the correlation between this and the Se content in the cell wall. For the direct determination of selenites in the medium, we applied a reaction with 2,3-diaminonaphthalene (DAN) for the precolumn derivatization of selenium for use in the reverse-phase high-performance liquid chromatography (RP HPLC) method. This method is based on the fact that DAN reacts only with SeO_3_^2−^ ions (selenites). Thus, the direct reaction of the medium sample with DAN (without mineralizing the sample) results in the selective determination of selenites. By contrast, the determination of total selenium content (all selenium compounds at different oxidation states) using RP HPLC requires the oxidation of Se(II) to Se(IV) during sample mineralization with HNO_3_. Heavy-metal cations (also Zn^2+^) may form complex compounds with selenites of higher stability than the selenite–DAN complex (piazselenol), which interferes with the aforementioned selenium(IV) determination method [26]. Therefore, in the determination method for total selenium, an EDTA masking agent is first added to the sample to remove this interference. Adding EDTA displaces selenite ions from complexes with heavy metals and allows them to form a selenite–DAN complex. Because we wanted to determine only “free” selenite ions in the culture medium (not bound in complexes with zinc), EDTA was not added to the sample before the determination. The direct determination of selenites in the culture media containing different molar ratios of Zn^2+^/SeO_3_^2−^ revealed that the selenite concentrations were much lower than the predicted values (Table 2). These results suggest that Zn(II) and selenite complexes formed in the medium have higher stability constants than 4,5-benzopiazselenol (selenite-DAN complex) because diaminonaphthalene had no effect on the displacement of selenites.

Note that the assumption of equivalence for the concentration of selenites not bound to DAN with the concentration of selenites complexed with zinc is undoubtedly true for experiments conducted in demineralized water when the absence of other factors that may interact with selenites is certain (e.g., by forming coordination bonds). There is no such certainty for the culture medium—there may be other components present, such that they may compete with zinc ions (cations of other metals, compounds containing amine nitrogen, etc.). Falsifying results is problematic in compounds that form complexes with selenites with higher stability than piazselenol; calculating the concentration of selenites complexed with zinc would then be burdened with an overstated error. Such an error, burdening the determination in the tested medium, is not significant, however, as indicated by the result of the determination of so-called “free selenites” for the culture medium enriched with 0.2 mM selenites: it is 0.202 mM. Therefore, the influence of selenite complexation with other factors is negligible and is within the determination error.

**Figure 10:**
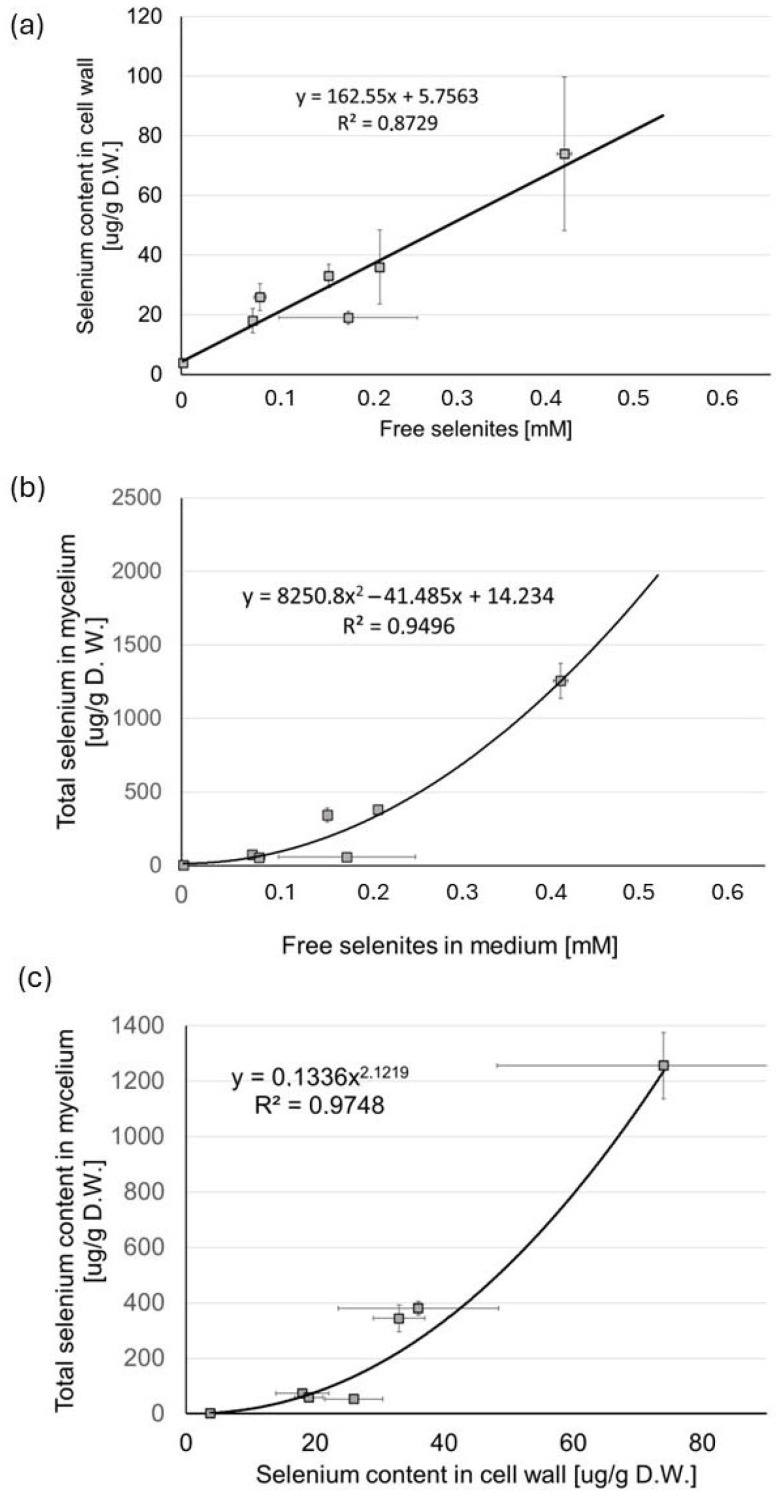
Correlation between (**a**) the concentration of selenites in the medium not bound in stable complex compounds (“free” selenites) and selenium content in the mushroom cell wall; (**b**) concentration of “free” selenites and total selenium content in the mycelium; (**c**) selenium content in the cell wall and total selenium content in the mycelium. All data represent the means of five experiments.

The correlation between the concentration of “free” selenites in the medium (not complexed with Zn(II)) and the selenium content in the mushroom cell walls (Figure 10a) was linear (R^2^ = 0.87). The total selenium content in mycelium (Figure 10b), as a function of the concentration of free selenites in the medium, was best fitted to a curvilinear polynomial expression (R^2^ = 0.95).

The linear dependence of selenium content in the cell wall on the selenite ion concentrations in the culture medium results from the mechanisms of the biosorption processes: the binding of selenite anions to the outer layer of the fungal cell wall, composed mainly of mannoproteins, can take place through both typical physicochemical interactions and the formation of chemical bonds, e.g., hydrogen or ionic. For low ion concentrations in the medium, these processes depend solely on this parameter. In turn, the nonlinear relationship between the concentration of selenites in the medium and selenium contents in the fungal biomass most likely results from the mechanisms of transport through the cell wall. The efficiency of the accumulation depends on the kinetics of two stages: the biosorption and the subsequent transport into the cell. Selenites enter mushroom cells primarily through phosphate transporters. Both high-affinity and low-affinity phosphate transporters are involved in this process. This process depends, among other things, on the concentration of phosphates: selenite and phosphate ions compete for these transporters. Therefore, the concentration of selenites in the culture medium is not the only factor determining the selenium content in the mycelium. To confirm that the nonlinear relationship between the selenite concentration in the culture medium and the selenium content in the biomass is the result of the kinetics of transport processes through the cell wall, we examined the correlation between the selenium concentration in the cell wall and its content in the fungal biomass (Figure 10c). In this case, the relationship was also nonlinear and best fitted to a curvilinear polynomial expression (R^2^ = 0.97).

## 3. Discussion

We attempted to obtain a new mushroom-derived preparation containing both selenium and zinc, two micronutrients necessary for the functioning of the immune system. This extended our interest in the simultaneous accumulation of these elements by *L. edodes* mycelia growing in media enriched with selenite and zinc(II) ions. Previously, we confirmed the feasibility of obtaining preparations isolated from selenium- and zinc-enriched *L. edodes* mycelium [29]. We examined preparations with different proportions of selenium and zinc on activating the T cell fraction of human peripheral blood mononuclear cells (PBMCs). We demonstrated that preparations with different proportions of polysaccharides, selenium, and zinc tended to upregulate the expression of all activation markers on both types of T cell populations stimulated in parallel with anti-CD3/anti-CD28 beads; however, statistically significant changes were observed only for PD-1 and CD25 antigens in CD8^+^ T cells. The selenium and zinc content in the examined preparations modified the immunomodulatory activity of mycelial polysaccharides, but the mechanisms of action of various active components of the mycelial extracts seemed to be different.

Thus, it is crucial to develop a method for obtaining preparations with an appropriate concentration of both zinc and selenium to achieve maximum activity in mycelium-derived preparations.

Previously, we found that the accumulation of selenium in the presence of zinc, and zinc in the presence of selenium, in the mycelial biomass significantly decreased compared with the number of individual ions absorbed when the medium is supplemented with that ion alone. Interestingly, the effect of the molar ratio of ions in the culture medium was more significant than the concentration value of each of them. The strongest proof comes from the results obtained when the mole fractions of XSeO_3_^2−^ in the SeO_3_^2−^/Zn^2+^ mixture were in a range of 0.33 to 0.5 (concentration ratio range: 1:2–1:1) [29]. We hypothesized that this phenomenon is caused by interactions between zinc(II) and selenites in the liquid medium, or more precisely, that the formation of zinc–selenite complexes most likely inhibits the first stage of the micronutrient accumulation process, i.e., biosorption. Therefore, the principal goal of the current study was to investigate how interactions between Zn^2+^ and SeO_3_^2−^ in an aqueous culture medium affect the biosorption and, in effect, accumulation of selenium and zinc in fungal cells. We excluded the effect of ZnSeO_3_ precipitation as the process responsible for reducing the effective ion concentration in the substrate, although zinc(II) and selenite may form zinc selenite, a salt partly soluble in water with a solubility product of K_spZnSO_3__ = 1.59 × 10^−7^ [59]. However, to avoid the precipitation of this salt in the culture medium, we selected ion concentrations low enough that the ion product (Q_sp_) would not exceed the solubility product (K_sp_) of this salt. Even at the highest concentrations used (0.2 mM and 0.8 mM), the ion product was only equal to K_sp_, so no zinc selenite precipitation in the culture medium was expected. However, zinc selenite precipitation is not the only possible interaction between selenite and zinc ions. According to the literature, in aqueous solutions, zinc(II) can form complexes with selenites, with different coordination numbers, stabilities, and charges [30,31]. Water molecules and—in a complex matrix such as a culture medium—other molecules may also participate in forming these complexes. The most common zinc(II) coordination numbers in these complexes are 4, 6, or 5 [60].

Our results obtained using the method of continuous variations (Job’s method) confirm that zinc(II) with selenites in aqueous solutions form complexes with stability and charge values dependent on their molar ratio. These complexes are most stable when the mole fraction of selenites in the mixture is in the 0.33–0.5 range, with a maximum of 0.4. The charge of the formed complexes is positive. Thus, the type of interactions these complexes have with the cell wall will probably be similar to the interaction of Zn(II) cations. This may be why the formation of complexes with the probable formula [Zn_3_(SeO_3_)_2_]^2+^ inhibits zinc biosorption less than that of selenites.

The molar fraction of selenites in the mixture with Zn(II) was equal to 0.4, at which point, zinc–selenite complexes of the highest stability form. However, does not fully coincide with the ratio of ion concentrations that most strongly inhibits the biosorption process. This ratio is 1:1, so the mole fraction of both ions is then equal to 0.5. The complex compounds formed in this case would have the general formula Zn_n_(SeO_3_)_n_ and be electrically neutral. The existence of such zinc and selenite complex compounds has been described. The obtained data show that the formation of negatively charged complexes inhibits the biosorption process of zinc ions more significantly than that of selenite ions; conversely, the formation of positively charged complex ions inhibits the biosorption of selenite ions more strongly. Hypothetically, the formation of complex ions with different charges influences ionic interactions with the external structures of the fungal cell wall. The biosorption process is the responsibility of the external layer of the fungal cell wall, a labile structure, with a composition dependent on the fungal species, the external conditions in which the fungus exists, stress factors, etc. In biotechnological cultivation methods, this depends on the composition of the culture medium, temperature, type of mixing, etc. The external layer of the fungal cell wall consists mainly of mannoproteins with functional groups typical of polysaccharides and proteins, e.g., hydroxyl, amino, or carboxyl groups [61,62]. The possibility of interaction between ions and mannoproteins results from their charge and structure, and they consist, for example, of ionic, hydrogen, van der Waals interactions, and adsorption processes. Therefore, the charge of the formed complex compounds or its absence significantly influences the formation of ionic interactions with the fungal cell wall. However, the lack of charge excludes the formation of strong ionic interactions, which significantly affects the biosorption process.

The results of directly determining non-complexed SeO_3_^2−^ ions in the culture media of different molar Zn(II)/selenites ratios confirmed that these ions could form complexes more stable than the product of the reaction between selenite and 2,3-diaminonaphthalene (4,5-benzopiazselenol).

The concentration of selenites in the medium not bound in stable complex compounds with Zn(II) correlated well with the selenium content in the mushroom cell wall.

As noted, depending on the molar proportions of these two ions, the complex compound could be charged positively or negatively or be neutral. The functional groups on the surface of the mycelial cell walls have their own charges and react with specifically charged ions as a reflection of their electrostatic character. The ability to form coordinative and hydrogen bonds with functional groups of the cell wall is most likely different for the zinc–selenite complexes than for separate zinc and selenite ions. These factors certainly impact the interactions between ions and the fungal cell wall, thus inhibiting biosorption and, as a result, bioaccumulation.

As stated in the Introduction, one of the objectives of our study was to optimize the conditions of simultaneous zinc and selenium accumulation in mycelial cultures of *L. edodes* to obtain a dietary supplement containing both selenium and zinc, two micronutrients necessary for the proper functioning of the immune system.

Our previous studies showed that the optimum concentration of sodium selenite in a cultivation medium—providing an *L. edodes* mycelium rich in organically bound selenium—was 0.12–0.25 mM (10–20 µg of Se/L) [26,27]. To provide the same selenium content in the mycelium when zinc (II) ions are present in the medium, the culture needs to be enriched with a substantially higher amount of sodium selenite (above 0.4 mM). At this concentration of selenites, the zinc ion contents should be lower than 0.4 mM to avoid zinc selenite precipitation. Considering the strong inhibitory effect of equimolar concentrations of selenites and zinc(II) on the accumulation of both elements, a zinc concentration of 0.2–0.3 mM is optimal. However, the amount of Zn in the mycelium cultured under these conditions will be much lower than in cultures supplemented with zinc salts exclusively.

## 4. Conclusions

We tested the hypothesis that the formation of zinc–selenite soluble complexes in a culture medium would strongly affect the first stage of ion transport into the cell, biosorption, and, thus, the accumulation of both elements (Zn and Se). In our opinion, the results are sufficient to confirm this. The method of continuous variations allowed us to find formulas for the most stable complex ions formed in the culture medium: [Zn_3_(SeO_3_)_2_]^2+^ and [Zn(SeO_3_)_3_]^4−^. However, further research is necessary on both the mechanisms of biosorption in the formed complexes and the influence of complexation on the transport of ions into the cell.

If optimizing the simultaneous accumulation of selenium and zinc in the mycelium of *L. edodes* does not lead to satisfactory results, there is another solution: the target preparation may be a mixture of selenium- and zinc-enriched *L. edodes* mycelium obtained separately, under the conditions we have previously developed.

## 5. Experimental Procedures

### 5.1. Microorganism and Cultivation Media

The *Lentinula edodes* (Berk.) Pegler strain used in this study was ATCC 48085. The seed culture was grown under conditions described in our previous work [6].

#### Supplementation of Media and Growth Conditions in Mycelial Shake-Flask Cultures

The liquid culture media contained glucose at 5%, yeast extract at 1%, soybean extract at 1.5%, and KH_2_PO_4_ at 0.1% (*w*/*v*). The pH of the medium was 6.5. Mineral precursors (sodium selenite and/or zinc bromide) were added to the medium, as shown in Table 3.

The zinc and selenium contents in the unenriched medium were the control levels in these studies.

Mycelia were grown in shake-flask cultures in 500 mL flasks containing 200 mL of medium. The fermentation medium was inoculated with 5% (*v*/*v*) of the seed culture. Cultures were incubated at 26 °C in a rotary shaker (New Brunswick Scientific, Edison, NY, USA) at 120 rev/min for 10 days. Mycelium was harvested via filtration, washed three times with redistilled water, and freeze-dried. All experiments were conducted five times to ensure reproducibility.

### 5.2. Isolation of Mycelial Cell Walls

Mycelial cell walls were prepared by modifying the method of Mahadevan and Tatum [63], as described by Schmit et al. [64]. One gram of lyophilized mycelium was ground and added to 100 mL of aqueous 1% sodium dodecyl sulfate. The mixture was stirred at 22 °C for 3 h followed by 15 h at 4 °C. The cell wall material was washed with distilled water six or seven times, lyophilized, and ground into a fine powder. This was followed by treatment with hot 80% ethanol (100 mL/500 mg of cell wall) for 20 min, filtration, and freeze-drying.

### 5.3. Determination of Selenium and Zinc in the Cultivation Medium, Cell Walls, and Mycelial Biomass

For the mycelium and isolated cell walls, determinations were performed after microwave mineralization.

#### 5.3.1. Mineralization Procedure

A microwave digestion procedure was performed using a closed-vessel microwave system (Magnum II, ERTEC, Knurów, Poland). A total of 0.02 g of sample was placed in a Teflon container and digested with 3 mL of 65% HNO_3_ (Suprapur quality, Merck, Darmstadt, Germany) in the microwave digestion system according to a four-stage program: 17–20 atm for 3 min (60% of the microwave power), 27–30 atm for 5 min (80% of the microwave power), 42–45 atm for 8 min (100% of the microwave power), and cooling for 10 min. When cool, the sample was diluted to 25 mL with deionized water. If necessary, the solutions were diluted several times. A blank digest was prepared in parallel.

#### 5.3.2. Determination of Zinc Content

Chromatographic analyses were performed as described previously [6] on a Dionex DX-500 ion chromatograph (Dionex, Sunnyvale, CA, USA) equipped with an IP 25 isocratic pump, an IonPac CG 5A guard column, an IonPac CS 5A analytical column (250 mm × 4.6 mm I.D., 9 µm bead diameter, ethylvinylbenzene functionalized with both quaternary ammonium and sulfonate functional groups), a 25 μL injection loop, and a Dionex AD20 absorbance detector with a Pneumatic Controller (PC 10) post-column reactor. The method described above was compared with an alternative chromatography (IC) determination procedure that provided comparable results.

#### 5.3.3. Determination of the Total Selenium Content

The reverse-phase high-performance liquid chromatography method with fluorimetric detection was used to determine selenium, as described previously [65]. The fluorescence was a linear function of the Se concentration in the tested range (correlation coefficient, R = 0.999). The RP HPLC conditions were as follows: eluent, acetonitrile; flow rate, 1.4 mL/min; temperature, 25 °C; injection volume, 20 μL. For fluorometric analysis, the excitation wavelength was 378 nm, and the emission wavelength was 557 nm. The piazselenol retention time was 3.1 min.

#### 5.3.4. Determination of Free Selenites in Cultivation Medium

In the cultivation media, free (not bound in stable complexes) selenites were directly determined, without mineralization [25].

### 5.4. Determination of the Composition and Stability of Zn(II)-Selenite Complexes in Liquid Culture Medium Using the Method of Continuous Variations (Job’s Method)

Stock solutions of sodium selenite (MilliporeSigma, 99%, Sigma-Aldrich, Saint Louis, MO, USA) and zinc bromide (MilliporeSigma, trace metal basis, Sigma-Aldrich, USA) were prepared at concentrations of 0.4 mM/L via dilution in deionized water (Merck, Darmstadt, Germany). Then, by mixing the stock solutions in the volume ratio provided in Table 4, a series of solutions containing the central ion and the ligand in different mutual ratios were prepared.

As a reference, two series of solutions containing only Zn^2+^ or SeO_3_^2−^ with decreasing concentrations in a range of 0.4–0 mM were prepared (Table 5).

Since the structures of the hypothetical complexes formed by selenites and zinc ions were unknown, the wavelength at which they showed maximum absorbance had to be determined experimentally. Wavelengths ranging from 200 to 700 nm were scanned using two complementary methods and a series of SeO_3_^2−^ and Zn^2+^ reference solutions (Table 5).

The first method involved a UV–Vis spectrophotometer, and the second method involved a liquid chromatograph with a diode array detector. The first method employed a Shimadzu UV Mini 1240 spectrophotometer.

The tests were performed by obtaining the UV–Vis spectra of samples measured in a quartz cuvette (High Precision Cell, QuartzSUPRASIL, Hellma Analytics, Müllheim, Germany) suitable for a 200–2500 nm range, with an optical path length of 10 mm. A high-quality vessel allowed us to perform short-wavelength-range scanning and absorbance measurements. The second method involved a Shimadzu UHPLC system consisting of the following modules: DGU-20A5 degasser, SIL-30AC autosampler, CBM-20A controller, CTO-20AC column oven, LC-30AD pumps, and SPD-M20A diode detector.

Tests were performed by injecting 40 µL of the samples into the system, which were then carried to the detector by 0.1 mL/min unaffected (without column) water flow. Each time, 90 s chromatograms and UV–Vis spectra were collected using a diode array detector (DAD), and the integral of the absorbance over time at a specific wavelength was measured. As a result of scanning the UV–Vis spectra using both methods, a range of 200–220 nm was selected for further tests, which were carried out for a series of wavelengths: 205, 210, 215, and 220 nm. The optimum for the experiments was found to be a wavelength of 215 nm, for which the absorbance was evidently dependent on the molar ratio of the ions in reference solutions. A series of isomolar selenite and zinc ion mixtures (Table 4) and corresponding reference solutions (Table 5) underwent tests using both methods. The first method was selected for further investigations. According to Job’s method, a graph of the absorbance versus composition of the solution (mole fraction of components) was plotted. The peak of the Job plot identifies the ligand-to-metal ratio at stoichiometric proportions and thus determines the formula of the coordination complex.

To consider the influence of other potential ligands originating from the components of the real liquid culture medium on the formation of zinc–selenite complexes, the experiment was repeated using stock solutions of SeO_3_^2−^ and Zn^2+^ ions in the real culture medium.

### 5.5. Statistical Analysis

Statistical analyses were performed using the STATISTICA data analysis software system, version 12, StatSoft, Inc. (2014, Tulsa, OK, USA). All computations were applied at a significance level of 0.05.

All data in groups were first tested for normality using the Shapiro–Wilk test. To compare the means of the two groups, a t-test was applied if the variances of the two populations were equal; otherwise, a Cochran–Cox test was applied. To check the homogeneity of variance, the Brown–Forsythe test was used.

Statistically significant differences between more than two groups were assessed using one-way analysis of variance preceded by checking the assumptions of normality and homogeneity of variance. For multiple comparisons, the Tukey post hoc test was performed, and to compare the control group to each of the others, Dunnett’s test was used.

## Figures and Tables

**Table 1:** Zn and Se contents in isolated mycelial cell walls and mycelial biomass (dry weight) versus Zn^2+^ and SeO_3_^2−^ molarity ratios in liquid culture medium.

Concentration of Medium Supplements		Zn^2+^/SeO_3_^2−^ Molarities Ratio	Cell Wall Composition		Reference Data: Biomass Composition ^#^	
Zn^2+^ [mM]	SeO_3_^2−^ [mM]		Zn [μg/g]	Se [μg/g]	Zn [μg/g]	Se [μg/g]
0	0	N/A	148.07 (20.27)	3.77 (0.62)	298.10 (29.02)	2.52 (0.17)
0	0.2	N/A	142.65 (10.28)	35.97 _a_ (12.43)	288.96 (50.83)	310.84 (84.59)
0.2	0	N/A	337.73 _b_ (59.48)	2.85 _b_ (0.91)	920.28 (152.99)	0.75 (0.28)
0.8	0.2	4:1	574.73 _c_ (44.74)	26.44 _c_ (4.47)	874.94 (194.16)	44.59 (6.76)
0.4	0.2	2:1	395.29 _b_ (72.57)	19.17 _c_ (2.27)	278.22 (33.31)	58.39 (12.65)
0.2	0.2	1:1	261.77 _b_ (18.93)	18.00 _b_ (4.11)	639.57 (45.95)	80.83 (4.16)
0.2	0.4	1:2	303.69 _a_ (72.32)	33.04 _a_ (3.99)	603.41 (24.20)	344.10 (48.65)
0.2	0.8	1:4	266.54 _c_ (8.36)	74.75 _b_ (25.75)	591.25 (18.41)	1395.89 (16.86)

All data represent the means of five experiments. Standard deviations (SDs) are noted in brackets. For the selenium and zinc content in the cell wall, the following significance levels were defined versus the reference culture grown in a medium not enriched with SeO_3_^2−^ and Zn^2+^: *p* < 0.05—a; *p* < 0.01—b, *p* < 0.001—c, and *p* > 0.05—no letter. ^#^ Data on the mycelial biomass composition are citations—they come from our previous publication [29].

**Table 2:** Concentration of selenites determined in medium via direct method (without mineralization) versus the theoretical value. All data represent the means of five experiments. Standard deviations (SDs) are noted in brackets.

Concentration of Medium Supplements		Zn^2+^/SeO_3_^2−^ Molarity Ratio	SeO_3_^2−^ Concentration [mM]	
Zn^2+^ [mM]	SeO_3_^2−^ [mM]		Determined	Theoretical
0	0	N/A	2 × 10^−4 a^ (10^−5^)	0
0	0.2	N/A	0.202 (3 × 10^−4^)	0.20
0.2	0.2	1:1	0.073 ^a^ (0.002)	0.2
0.4	0.2	2:1	0.173 ^a^ (0.003)	0.2
0.6	0.2	3:1	0.114 ^a^ (0.002)	0.2
0.8	0.2	4:1	0.078 ^a^ (0.005)	0.2
0.2	0	N/A	0.0002 ^a^ (3 × 10^−5^)	0
0.2	0.2	1:1	0.073 ^a^ (0.002)	0.2
0.2	0.4	1:2	0.152 ^a^ (0.001)	0.4
0.2	0.6	1:3	0.279 ^a^ (0.005)	0.6
0.2	0.8	1:4	0.390 ^a^ (0.007)	0.8

^a^ Level of significance versus reference culture cultivated in medium rich in 0.2 mM SeO_3_^2−^, *p* < 0.001.

**Table 3:** Supplementation protocol for culture media.

Precursor	Concentration of Precursor [mM]							
SeO_3_^2−^	0	0.2	0	0.2	0.2	0.2	0.4	0.8
Zn^2+^	0	0	0.2	0.8	0.4	0.2	0.2	0.2

**Table 4:** Preparation of a series of isomolar mixtures of selenite and zinc ion stock solutions.

Ion	0.4 mM Volume Stock Solution (mL)										
SeO_3_^2−^	0	1	2	3	4	5	6	7	8	9	10
Zn^2+^	10	9	8	7	6	5	4	3	2	1	0
	**Ion mole fraction (X)**										
SeO_3_^2−^	0	0.1	0.2	0.3	0.4	0.5	0.6	0.7	0.8	0.9	1
Zn^2+^	1	0.9	0.8	0.7	0.6	0.5	0.4	0.3	0.2	0.1	0
	**Ion concentration (mM/L)**										
SeO_3_^2−^	0	0.04	0.08	0.12	0.16	0.2	0.24	0.28	0.32	0.36	0.4
Zn^2+^	0.4	0.36	0.32	0.28	0.24	0.2	0.16	0.12	0.08	0.04	0

**Table 5:** Preparation of a series of SeO_3_^2−^ and Zn^2+^ reference solutions.

Component	Volume (mL)										
0.4 mM SeO_3_^2−^ or Zn^2+^	0	1	2	3	4	5	6	7	8	9	10
H_2_O	10	9	8	7	6	5	4	3	2	1	0
	**Final concentration (mM)**										
SeO_3_^2−^ or Zn^2+^	0	0.04	0.08	0.12	0.16	0.2	0.24	0.28	0.32	0.36	0.4

## Data Availability

The data presented in this study are available on request from the corresponding author and co-authors.
